# Pathological markers of Piezo1 at distal anastomosed ureter in predicting pyeloplasty outcomes in children

**DOI:** 10.3389/fped.2025.1612706

**Published:** 2025-10-28

**Authors:** Jincai Zhou, Xun Lu, Liqu Huang, Yunfei Guo

**Affiliations:** ^1^ Department of Urology, Affiliated Jianhu Hospital of Xinglin College, Nantong University, Nantong, China; ^2^ Department of Urology, Children’s Hospital of Nanjing Medical University, Nanjing, China

**Keywords:** Piezo1, pathological markers, distal ureter, predictive value, pyeloplasty outcomes

## Abstract

**Objective:**

To evaluate the prognostic value of Piezo1 expression in the distal anastomosed ureter for predicting pyeloplasty outcomes.

**Methods:**

Distal anastomosed ureter specimens from 28 patients diagnosed with primary ureteropelvic junction obstruction (UPJO) undergoing laparoscopic pyeloplasty were analyzed. Immunohistochemistry assessed Piezo1 expression, and Masson's trichrome staining determined the collagen-to-muscle ratio (CM ratio). Postoperative outcomes at 12 months were categorized as excellent improvement, good improvement, mild improvement, no improvement, or deterioration based on hydronephrosis severity scores (HSS). Associations between pathological markers (Piezo1 and CM ratio) and surgical outcomes were examined.

**Results:**

The cohort included 28 patients (22 male, 6 female), with a median age of 64.3 months (IQR: 15.4–102.6). Compared to the unsatisfactory outcome group (no improvement/deterioration), the satisfactory group (excellent/good/mild improvement) exhibited significantly lower Piezo1 expression and CM ratio in the distal anastomosed ureter. Piezo1 expression strongly correlated with CM ratio (*r* = 0.687, *p* < 0.0001). Piezo1 expression level threshold of <2.765% predicted satisfactory outcomes.

**Conclusion:**

Distal anastomosed ureter Piezo1 expression is a promising prognostic biomarker for pyeloplasty outcomes.

## Introduction

Ureteropelvic junction obstruction (UPJO) is the most common etiology of hydronephrosis in pediatric populations, with an estimated global incidence of 1:1500 (1–3). Since its initial introduction in 1949, the Anderson-Hynes dismembered pyeloplasty has remained the gold-standard reconstructive procedure for pediatric UPJO (4). Although the surgery demonstrates high success rates, with reported failure rates of approximately 5% (5–7), the precise pathogenesis of UPJO remains unclear. In clinical practice, surgeons routinely submit resected stenotic segments for histopathological evaluation; however, findings are typical fibrosis and inflammatory cell infiltration with limited prognostic value (8).

Previous studies have demonstrated that interstitial cells of Cajal (ICC) play a crucial role in modulating ureteral peristalsis, with reduced ICC density at the UPJ being implicated in the pathogenesis of hydronephrosis (9–11). Additionally, characteristic histopathological features of UPJO include smooth muscle hypertrophy, excessive collagen deposition, and neuron deficiency (12–14). While existing research has primarily focused on UPJ specimens to elucidate UPJO etiology, the prognostic significance of pathological markers in the resected distal ureter remains poorly understood.

The Piezo-type mechanosensitive ion channel component 1 (Piezo1) is a mechanotransduction protein that gates in response to mechanical stimuli, transducing physical forces into electrochemical signals to modulate diverse physiological processes including vascular tone regulation, cellular differentiation, and urinary tract dynamics (15, 16). Immunohistochemical studies have identified Piezo1 localization in urothelial cells, ureteral smooth muscle, and renal pelvis, where it contributes to ureteral peristalsis modulation (17). However, the expression profile of Piezo1 in human ureter remains elusive, and its potential association with pyeloplasty outcomes has not been investigated.

This study aimed to characterize Piezo1 expression patterns in distal ureteral tissues and evaluate its potential as a predictive biomarker for pyeloplasty outcomes.

## Materials and methods

### Study design

This retrospective study was conducted at the Children's Hospital of Nanjing Medical University from September 2022 to November 2023, with approval from the Institutional Review Board (Approval number: 202503017-1). All procedures complied with the Declaration of Helsinki principles, and written informed consent was obtained from all participants' legal guardians. The study included pediatric patients who underwent laparoscopic pyeloplasty for primary UPJO. Exclusion criteria included: (1) concurrent renal malformation (duplex kidney, solitary kidney, horseshoe kidney, or ectopic kidney); (2) bilateral UPJO; (3) vesicoureteral reflux; (4) secondary procedures or crossing vessel for UPJO; and (5) incomplete clinical data or lost in follow-up. The schedule for routine follow-up was at 3, 6, 12, 18 and 24 months after surgery under outpatient or with telephone interview.

### Treatment

All patients underwent standardized assessment including renal ultrasonography for measurement of anteroposterior diameter (APD) and cortical thickness evaluation. Diuretic renography was performed to assess differential renal function (DRF) and urinary drainage patterns. Surgical indications included: (1) progressive hydronephrosis (Society for Fetal Urology grade III-IV) on ultrasonography; (2) presence of clinical symptoms (renal colic, urinary tract infections, hematuria, or nephrolithiasis); and (3) impaired renal function (DRF <40%) or obstructive drainage pattern (T_1/2_ >20 min post-furosemide administration). All surgical procedures were performed by three experienced surgeons with the same qualifications in laparoscopic surgery. Baseline characteristics including patient's age, gender, side, weight, diagnosis, APD and differential renal function (DRF) were recorded preoperatively.

### Pathological analysis

The collected distal anastomosed ureter samples were fixed with 10% buffered formalin and embedded by paraffin. Then, paraffin-embedded samples were sectioned into 4-*μ*m thickness for H&E staining. For Masson's trichrome staining, sections were stained following conventional Masson's trichrome, which indicates the collagen as blue and smooth muscle as red. The collagen to muscle ratio (CM ratio) was analyzed according to Kim et al (18). Immunohistochemical for evaluation the expression of Piezo1 were conducted by incubating with H_2_O_2_ before treatment with anti-Piezo1 antibody (1:100, proteintech, China). The positive areas of piezo1 were measured under a light microscope. All histopathological assessments were conducted in a blinded manner.

### Definition and outcomes

In present study, pyeloplasty outcomes after surgery were defined based on hydronephrosis severity score (HSS), which was proposed by Babu et al (19). Generally, HSS was calculated by three parameters including DRF, drainage curve pattern, and US grade. Each item was scored from 0 to 4 points. Thus, the total HSS ranged from 0 to 12 points (Table 1). Finally, pyeloplasty outcomes were defined as excellent improvement, good improvement, mild improvement, no improvement and deterioration at 12 months after surgery based on the difference between preoperative and postoperative HSS values. Patients presented with excellent improvement, good improvement, mild improvement were classified as satisfactory group; while patients presented with no improvement and deterioration were defined as unsatisfactory group.

**Table 1:** Hydronephrosis severity score is determined by three parameters: differential renal function (DRF), drainage pattern and ultrasound grade/anteroposterior diameter (APD).

Hydronephrosis severity score = A + B + C			
Score	A. DRF	B. Drainage pattern	C. APD
0	≥45%	Good drainage starts even before frusemide	≤5 mm
1	40–44%	Good drainage starts only after frusemide	6–9 mm
2	35–39%	Delayed drainage after frusemide (equivocal)	10–19 mm
3	30–34%	Poor response to frusemide (plateau) + partial clearance in 2-h	20–29 mm
4	<30%	No response to frusemide (up-raising curve) + stasis in 2-h	≥30 mm

### Statistical analysis

Numerical variables were presented as mean ± standard deviation (SD) or median with interquartile range (IQR), depending on their normality distribution. Accordingly, the *t*-test or Mann–Whitney *U*-test was conducted as appropriate. Categorical variables were expressed as frequencies with percentages and were compared using the chi-square test or Fisher's exact test. The positive areas of Piezo1 in distal ureter were measured under a light microscope and calculated by ImageJ software (version 1.51, USA). The prognostic potential of Piezo1 was determined using receiver operating characteristic (ROC) curve analysis, with results expressed as the area under the curve (AUC). The optimal cutoff value was identified based on the combined consideration of sensitivity and specificity. The ROC curves were constructed using MedCalc software (version 20.015). All statistical analyses in this study were performed using Stata (version 15.1), with a *p*-value <0.05 considered statistically significant.

## Results

### Baseline characteristic

Overall, 28 patients were included in present study. The median age of the entire population at surgery was 64.3 (IQR: 15.4–102.6) months with 22 (78.6%) male patients and 6 (21.4%) female patients. The median weight was 19.5 (IQR: 10.0–32.8) kg, and 22 patients (78.6%) was confirmed in left side. The median pre-APD and DRF were 32.5 (IQR: 23.5–45.5) mm and 43.6 (IQR: 32.1–46.7), respectively. Most patients (82.1%) were prenatally diagnosed with hydronephrosis. The median follow-up duration was 16 (IQR: 12–18) months. Detailed patients' characteristics were shown in Table 2.

**Table 2:** Baseline characteristics of included patients.

Variable	Overall (*n* = 28)
Gender	
Male	22 (78.6%)
Female	6 (21.4%)
Side	
Left	22 (78.6%)
Right	6 (21.4%)
Age, months	64.3 [15.4–102.6]
Weight, kg	19.5 [10.0–32.8]
Diagnosis	
Prenatally	23 (82.1%)
Postnatally	5 (17.9%)
Pre-APD, mm	32.5 [23.5–45.5]
DRF, %	43.6 [32.1–46.7]

### Clinical outcomes

Table 3 represented the clinical outcomes. The results showed that 8 patients were excellent improvement, 11 patients were good improvement, and 4 patients were mild improvement. However, two patients presented with no improvement and they were still under follow-up. There were 3 patients presented with deterioration and all received a repeated pyeloplasty.

**Table 3:** Clinical outcomes and quantitative analysis of pathological markers of included patients.

Clinical outcome	Pre HSS-Post HSS	*N* = 28	Piezo 1 area (%)	CM ratio
Excellent improvement	5–6	8	1.768	0.772
Good improvement	3–4	11	1.602	0.786
Mild improvement	1–2	4	1.834	0.714
No improvement	0	2	3.158	1.900
Deterioration	−1 or less	3	3.525	1.871

### Pathological outcomes

**Figure 1:**
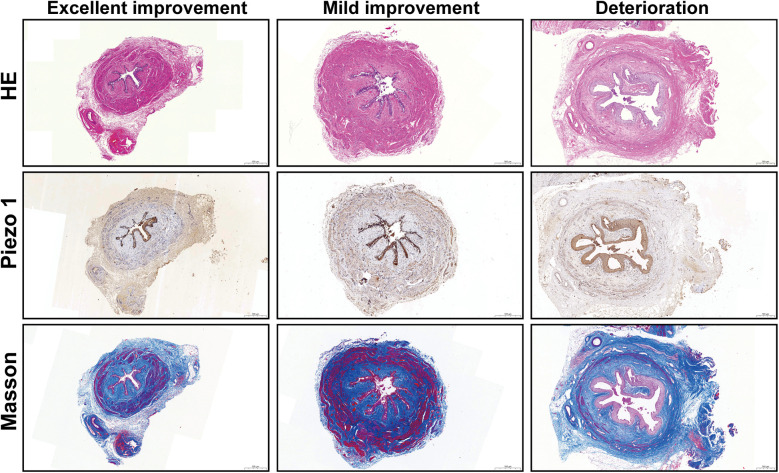
Representative HE, Piezo1 and Masson staining images in distal ureter (scale bar, 500 μm).

**Figure 2:**
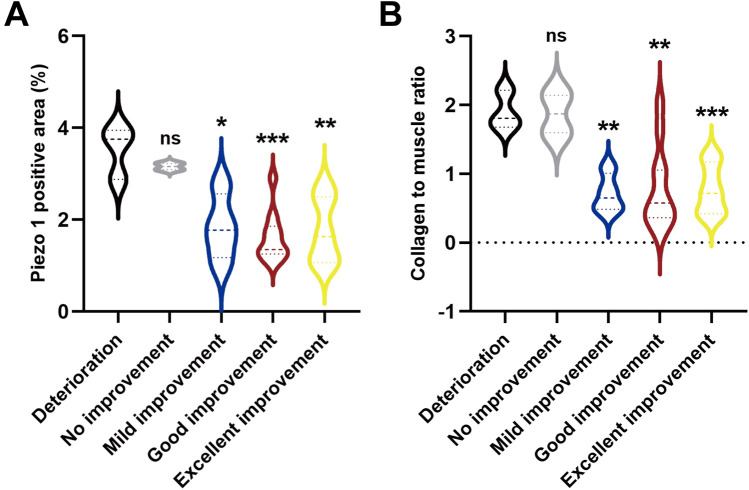
**(A)** Quantitative analysis of Piezo1 positive areas in distal ureter. **(B)** Quantitative analysis of CM ratio in distal ureter. (*P < 0.05, **P < 0.01, and ***P < 0.001 vs. deterioration group).

The representative images were shown in Figure 1. The median positive area of piezo1 at distal ureter was significantly lower in excellent improvement (*p* = 0.004), good improvement (*p* < 0.001) and mild improvement (*p* = 0.021) compared to deterioration group. While no significant difference was found between no improvement and deterioration group (3.525 vs. 3.158; *p* = 0.455). Additionally, the CM ratio was significantly lower in excellent improvement (*p* < 0.001), good improvement (*p* = 0.010) and mild improvement (*p* = 0.003) compared to deterioration group at distal ureter. And there was no significance difference between no improvement and deterioration group (1.871 vs. 1.900; *p* = 0.927) (Figure 2).

### Prognostic potential of Piezo1

**Figure 3:**
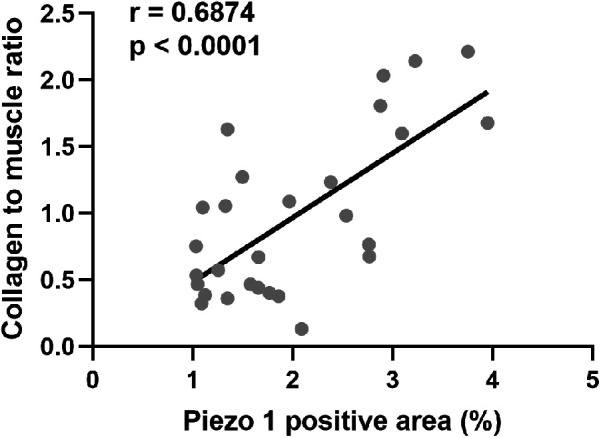
The correlation between Piezo1 positive area and CM ratio.

To explore the clinical implications of Piezo1, the correlation analysis between Piezo1 and CM ratio in distal ureter was conducted. The results indicated that piezo1 expression had a good correlation with CM ratio (*r* = 0.687; *p* < 0.0001) (Figure 3).

**Figure 4:**
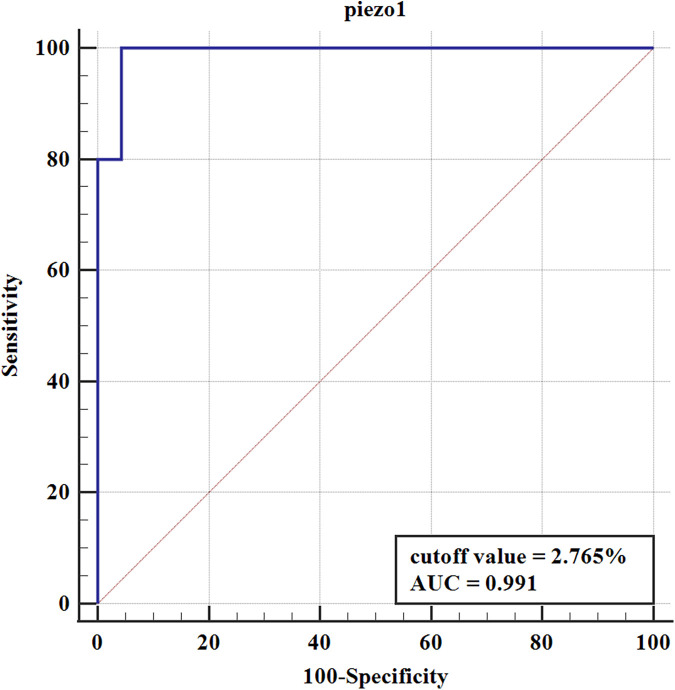
The ROC of Piezo1 in predicting pyeloplasty outcomes.

To further elucidate the prognostic value of Piezo1 in pyeloplasty outcomes, the ROC curve was constructed. Correspondingly, the pyeloplasty outcomes were classified as satisfactory group (excellent improvement, good improvement, and mild improvement) and unsatisfactory group (no improvement and deterioration) according to HSS. The ROC curve was shown in Figure 4. The results suggested that Piezo1 had a high predictive value for pyeloplasty outcomes, with an area under the curve (AUC) of 0.991. The best cutoff value of Piezo1 was 2.765%, with the sensitivity of 100.00% and the specificity of 95.65%.

## Discussion

While pyeloplasty is generally successful in treating UPJO, researchers have sought to identify histological markers in predicting surgical outcomes. Previous studies have demonstrated the prognostic significance of ICC, neuron density, and CM ratio at the UPJ (10, 12, 14). In this study, we investigated Piezo1 expression in the distal ureter and demonstrated its potential as a predictive biomarker for pyeloplasty outcomes.

Yang et al. (10) reported a significant reduction in ICC density within UPJ samples compared to controls, suggesting a potential role for ICC in UPJO pathogenesis. Additionally, Senol et al. (11) pointed that the number of ICC was decreased in most UPJO cases, which was similar to the study reported by Inugala et al. (20) in which 52% of UPJO patients were defined as negative of ICC. While the direct evidence between ICC and ureteral motility is insufficient, previous studies have demonstrated that a wide range of ion channels and receptors were expressed on ICC, which were involved in modulating smooth muscle cells contraction (21).

The relationship between histopathological features and pyeloplasty outcomes remains controversial. Issi et al. (22) conducted a retrospective study including 52 patients and reported that there were not any significant differences in collagen type 3, elastin, fibrosis, or ICC between successful and unsuccessful surgery group. While some authors failed to demonstrate the association between pathological markers and outcomes and they claimed that surgical techniques were more important, we believe that they missed the association because they only focused on the UPJ segment itself. It is essential to comprehensively analyze the resected distal ureter as it was used for anastomosis and determines the long-term outcomes.

Babu et al. (23) demonstrated that pathological parameters at the anastomotic ureteral end correlate more strongly with clinical outcomes than those at the UPJ. Their findings indicated that, compared to ICC and neurons, the CM ratio at the anastomotic site serves as a superior predictor of pyeloplasty success. This was further supported by Kim et al. (18), who confirmed the prognostic value of CM ratio in assessing post-pyeloplasty recovery. Additionally, Ulusoy et al. (24). Indicated that renal pelvis collagen ratio was significantly increased in patients with severe renal function loss and stated that analysis of renal pelvis collagen ratio would help to evaluate renal function in advance in congenital hydronephrosis. Our study revealed that Piezo1 is predominantly expressed in the distal ureter and shows significant positive correlation with the CM ratio (*r* = 0.687, *p* < 0.0001). Importantly, Piezo1 expression levels at the anastomotic site effectively predicted surgical outcomes. This finding aligns with established literature documenting Piezo1's involvement in fibrotic processes in liver and kidney tissues (25, 26). Through receiver operating characteristic (ROC) analysis, we identified ≤2.765% Piezo1-positive area as a predictive threshold for successful pyeloplasty outcomes. While routine histological examination of UPJ specimens typically reveals only typical fibrosis and inflammatory cells, quantitative assessment of Piezo1 expression in the anastomotic ureteral segment may provide clinically meaningful prognostic information.

While immunohistochemistry for Piezo1 may not be widely available or affordable, we recommend routine Masson's trichrome staining with quantitative CM ratio analysis as a practical alternative. Our findings strongly support the prognostic value of distal anastomosed ureteral Piezo1 expression assessment in predicting pyeloplasty outcomes. The results in present study have important clinical implications as patients demonstrating unfavorable histopathological characteristics in the distal ureter may benefit from more intensive postoperative surveillance, and prolonged DJ stent retention could be considered in such cases to potentially reduce recurrence risk. This stratified approach based on histopathological risk assessment may optimize postoperative management while maintaining cost-effectiveness.

It has several limitations that should be acknowledged. First, the study is limited by its retrospective nature and relative small numbers. Second, the exclusion of patients with crossing vessels introduces potential selection bias, warranting specific investigation of this subgroup in future studies. Third, while we identified Piezo1 expression as a predictive biomarker for pyeloplasty outcomes, the precise molecular mechanism remains unclear. Additional cellular and animal studies are needed to elucidate the underlying molecular pathways involving Piezo1 in ureteral function and repair. Further research should pay more attention to developing intraoperative markers to help identify normal ureters by frozen sections or special staining during pyeloplasty.

## Conclusion

Distal anastomosed ureter Piezo1 expression is a promising prognostic biomarker for pyeloplasty outcomes.

## Data Availability

The raw data supporting the conclusions of this article will be made available by the authors, without undue reservation.
